# Prevalence of Head and Neck Tumors in Children under 12 Years of Age Referred to the Pathology Department of Children’s Hospital in Tabriz during a 10-year Period

**DOI:** 10.15171/joddd.2015.019

**Published:** 2015-06-10

**Authors:** Shirin Fattahi, Sepideh Vosoughhosseini, Monir Moradzadeh Khiavi, Seyed Mostafa Mahmoudi, Parya Emamverdizadeh, Seyed Gholamreza Noorazar, Neda Yasamineh, Rana Lotfi

**Affiliations:** ^1^Assistant Professor, Department of Oral and Maxillofacial Pathology, Faculty of Dentistry, Tabriz University of Medical Sciences, Tabriz, Iran; ^2^Professor, Department of Oral and Maxillofacial Pathology, Faculty of Dentistry, Tabriz University of Medical Sciences, Tabriz, Iran; ^3^Associate Professor, Department of Oral and Maxillofacial Pathology, Faculty of Dentistry, Tehran University of Medical Sciences(International Cam-pus), Tehran, Iran; ^4^Assistant Professor, Department of Oral and Maxillofacial Pathology, Faculty of Dentistry, Birjand University of Medical Sciences, Birjand, Iran; ^5^Assistant Professor, Department of Oral and Maxillofacial Pathology, Faculty of Dentistry, Tabriz University of Medical Sciences, Tabriz, Iran; ^6^Assistant Professor, Clinical Psychiatry Research Center, Department of Psychiatry, Tabriz University of Medical Sciences, Tabriz, Iran; ^7^Postgraduate Student, Department of Prosthodontics, Faculty of Dentistry, Tabriz University of Medical Sciences, Tabriz, Iran; ^8^Life science student, Arts & Science Faculty, University of Toronto, Toronto, ON, Canada

**Keywords:** Head and neck tumor, children, benign tumors, malignant tumors

## Abstract

***Background and aims.*** Head and neck tumors are the most common complaints of people referring to different medical sections, especially in children. The aim of this study was to evaluate the prevalence of these tumors in children less than 12 years of age to provide a better perspective for future studies.

***Materials and methods.*** All the files in Department of Pathology at Tabriz Pediatric Hospital from 2001 to 2011 were screened for head and neck tumors in children under 12 years of age. Data including age and gender as well as the type, the location, and benign/malignant characteristic of the tumor were recorded. Data were analyzed by SPSS 15 statistical software, using descriptive statistics and chi-square test.

***Results.*** A total of 160 cases were identified. Most of the tumors were benign (68%) and most of the tumors occurred in the neck region (41%). The most frequent benign and malignant tumors were lymphangioma and non-Hodgkin lymphoma, respectively. The majority of benign tumors were found in children younger than 2 years old (P=0.007), but there was no age predilection for malignant tumors.

***Conclusion.*** According to our results, benign tumors were more prevalent than malignant ones. Although a low rate of benign tumors in males shows that more attention should be paid to the early diagnosis of head and neck tumors.

## Introduction

General and pediatric dentists should have enough knowledge of the diagnosis and treatment of oral diseases in children during oral examination in order to provide appropriate medical care for them.^1-2^ Tumors are tissue masses that are divided into benign and malignant categories, according to their biological behavior.^3^Tumors may occur at different ages and with different tissue origins. Delay in providing care can sometimes lead to morbidity, even in the case of benign tumors with a favorable prognosis.^4^Malignant tumors of head and neck in children might result in high rates of morbidity and mortality and are considered the second most important etiologic factor for mortality in 4-15-year-old children.^5^ The annual increase in cancer rates children has been reported to be 1-2% in different countries. However, data on prevalence of head and neck tumors is scarce.^5^

A study by Rapidis et al^6^ on 1007 child patients with one type of head and neck tumor showed that 30.6% of the tumors were malignant, 27.8% were benign, and the rest included tumor-like conditions and dysplasias from embryonic remnants. In this study, lymphoma was the most common malignant tumor and hemangioma was the most common benign tumor; lymphoma was common among 3-8-year-old males and 6-10-year-old females; and hemangioma was prevalent in children 5 years of age with equal predilection for both sexes.^6^Another study by Asamoa et al^4^ on 1340 child patients showed that non-odontogenic and mesenchymal tumors were the most common tumors with an age average of 8.7 years and often in males. According to the study of Sengupta et al^7^ on children under 12 years of age who had head and neck tumors, the most common malignant tumors were lymphoma, rhabdomyosarcoma and nasopharyngeal carcinoma, in descending order; and non-Hodgkin lymphoma was the most common among lymphomas. Overall, all the malignant lesions were more prevalent in children over 5 years of age (69.81%), especially in 10-12-year-olds.^7^ Benign tumors in children’s head and neck region are not common but can include tumors originating from thyroid tissues, salivary glands, the nervous system, the adipose tissue, and the bones.^8^ Because of the complicated anatomy, it is difficult to provide suitable clinical treatment for neoplasms of the head and neck region in children.^6^

Due to the importance of epidemiological studies in different geographical regions, we attempted to collect demographic data on the head and neck tumors in children under 12 years of age from Children's Hospital in Tabriz, in the North-west of Iran. The data on incidence of benign and malignant tumors during a 10-year period can provide a better understanding of the current situation for future studies.

## Materials and Methods

In this retrospective descriptive-analytical study, the patient records at the Department of Pathology at Tabriz Children's Hospital during the period from 2001 to 2011 were evaluated. The files of patients under 12 years of age with head and neck tumors were included in this study. Only files with the final pathological diagnosis of a head and neck tumor were included. Exclusion criteria consisted of records with no exact histological diagnosis, those involving a proliferative lesion with an inflammatory or allergic origin such as the adenoid and nose polyp, tumor-like lesions, and congenital/developmental malformation or cystic lesions.

Data were collected from the files and recorded in a special form that included the demographic data of the patients including age and gender, and information on the kind of tumor and its location, the biological behavior of the tumor (benign or malignant), and the tissue origin of the tumor (epithelial or mesenchymal). Data were analyzed with SPSS 15 software using descriptive statistics (means and standard deviations) and chi-square test. Chi-square test was used to evaluate the relationship between tumor behavior and the gender as well as the tissue of origin.

## Results

From a total of 2158 records involving head and neck lesions, 149 records matched the inclusion/exclusion criteria of the study. In 11 records, the tumor was seen in two locations and therefore, the total number of tumors was 160. The minimum age of the studied patients was 2 days and the maximum age was 12 years, with a mean of 5.2 ± 3.9 years.

**Figure 1.**
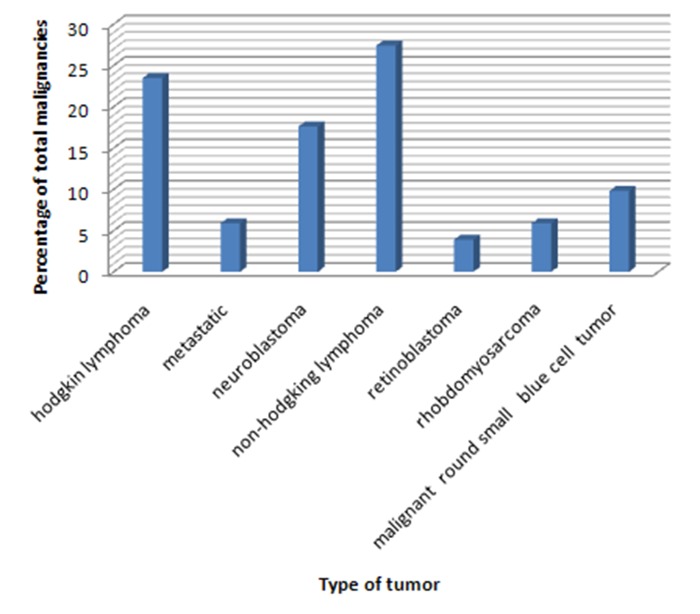
The distribution of malignant tumors in the studied period.

**Figure 2.**
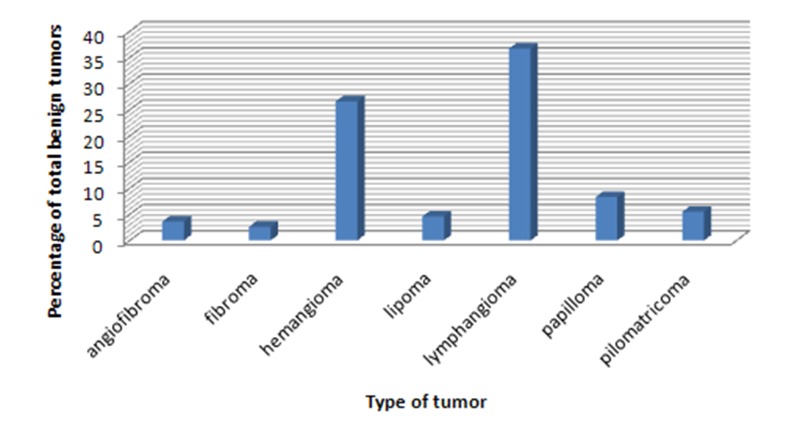
The distribution of benign tumors in the studied period.

The most common locations for tumors, in descending order, were neck (41%), head (8.1%), nose (6.25%), and the tongue (5.6%). 109 tumors were benign (68%) and 51 were malignant (32%). The most common benign and malignant tumors were lymphangioma and non-Hodgkin lymphoma, respectively (Figures 1 & 2). The majority of benign tumors were seen in children younger than 2 years of age, but no such attribution to a special age was noted in the case of malignant tumors. In addition, distribution of lymphangioma was the same in both genders. Table 1 presents the prevalence of benign and malignant tumors according to gender. Chi-square test showed no relationship between tumor behavior and gender. The overall female-to-male ratio was 1:1.25 (P = 0.098). The most common tumors in both genders are presented in Table 2. The tumor behavior showed no relationship with the tissue of origin (P = 0.008; Table 3).

**Table 1:** Incidence of tumors (gender and type)^*^

**Gender**	**Benign**	**Malignant**	**Total**
**Girl**	53	17	70
**Boy**	54	34	88
**Total**	107	51	158
P=0.098			
^*^Two records failing to indicate gender are not included.			

**Table 2:** The most common tumors (frequency more than 4) based on gender

**Gender**	**Tumor**
**Girl**	**Lymphangioma, hemangioma, papilloma**
**Boy**	**lymphangioma-hemangioma- non-Hodgkin lymphoma-hodgkin lymphoma –neuroblastoma**
P=0	

**Table 3:** Tissue source distribution of tumors (benign and malignant)^*^

**Tissue source**	**Malignant**	**Benign**	**Total**
**Mesenchymal**	47	90	137
**Epithelial**	1	19	20
**Total**	48	109	157
P=0.008			
^*^Three records of metastatic tumors failing to indicate the exact tissue origin are not included.			

## Discussion

In this study, head and neck tumor incidence in children under 12 years of age who referred to a children’s hospital was evaluated. The results showed that the most common benign and malignant tumors were lymphangioma and non-Hodgkin lymphoma.

Lymphangioma is manifested in head and neck region in 50‒75% of the cases, and in the oral region, the most common location is the anterior two-thirds of the tongue; about half of all lesions are noted at birth.^8^ Non-Hodgkin lymphoma often appears in adults, but children are also affected; and it is often extra-nodal in the oral region.^8,9^ Rapidis et al^6^ evaluated 1007 children with head and neck tumors, and according to the results, 30.6% of tumors were malignant and 27.8% were benign. Lymphoma and hemangioma were the most common tumors among malignant and benign tumors, respectively.^6^ Differences are in proportions of benign and malignant tumors and in most common benign tumor between the two studies. However, the results are consistent in relation to malignant tumors. Such differences might be attributed to geographical location, genetic factors, epidemiological differences of the regions and also the sample sizes. In another study on 134 patients, non-odontogenic and mesenchymal origins of tumors were seen most commonly.^4^ There is similarity in the tissue origins of the tumors and the most commonly affected gender between these results and ours. Agreement with another study in Niger can be noted in head and neck malignant tumors in children and teenagers, with boys being more commonly affected.^10^ In addition, based on a study in 1974 on head and neck tumors of children, soft tissue tumors of the orofacial region were the most common benign tumors, followed by tumors in the jaws.^11^ Also, the ratio of benign tumors to malignant tumors in children was 10:1, with higher prevalence in girls.^11^ In comparison with the latter study, there is a similarity in relation to the dominance of benign tumors, with differences in gender and the most commonly affected location. The discrepancies might be attributed to differences geographic location and therefore epidemiologic differences of the regions. A previous study on children under 12 with had head and neck tumors, the most common malignant tumors were lymphoma and among lymphomas, non-Hodgkin lymphoma was more prevalent.^7^ Overall, all the malignant lesions were more prevalent in 5-year-old children.^7^ The most common malignant tumor is the same for the latter study and ours; however, the two studies cannot be compared due to lack of data on age ranges. Non-Hodgkin lymphoma has also been noted as the most common malignant tumor in the head and neck region in children.^12^

Tanrikulu et al^13^ found most of the maxillofacial tumors in 11-15-year-old children with the mandible odontogenic and non-odontogenic tumors comprising 23.3% and 76.7% of tumors, respectively. Tumors with mesenchymal origin were the most common non-odontogenic tumors, and there were no significant differences in sex predilection.^13^ Comparison of sex dominance between this study and ours shows almost the same statistical ratios. In addition, in relation to tissue origin, mesenchymal tumors were the most common in both studies, with no differences in the location. In the latter study, the maxillofacial region was evaluated, while in ours, all the head and neck region was considered. Another study on the head and neck tumors in children younger than 16 years old in Ghana found 30.3% and 69.7% of tumors to be malignant and benign, respectively.^14^ The most malignant tumor was lymphoma and the most common benign tumor was squamous papilloma.^14^ The most common locations for benign and malignant tumors were the larynx and neck, respectively.^14^ Consistent with our study, benign tumors were more prevalent than malignant tumors, with a predilection for boys in both studies. The most common malignant tumor was the same in both studies, with a difference for benign tumors.

In a retrospective study by Cunningham et al^15^ on the head and neck malignant tumors, 241 patients under 19 years of age with head and neck malignant tumors were studied. Hodgkin lymphoma and other lymphomas were found to be more prevalent, and the nasopharynx, orbital fossa, face, scalp and salivary glands were the most common locations for malignant tumors, respectively.^15^ The most common malignant tumor was the same as in our study. In the study of Tanaka et al^16^ on 105 children under 15 years of age with head and face tumors, 97.1% were found to be benign. With regards to the dominance of benign tumors, the results of both studies are consistent but with differences in ratios, which might be attributed to different age ranges. In a study by Albright et al^5^ on 3050 head and neck tumors in children, the most common malignant tumor was lymphoma. The most common location was the neck,^5^and the most common malignant tumor, its location, and gender dominance were the same as the present study.

A major limitation of the present study was the inadequate access to all patients referring to health centers and some cases of inaccurate data in patient records, resulting in exclusion from the study.

## Conclusion

In the evaluated records, the most common location of tumors was the neck region, and the majority of head and neck tumors were benign. The most common benign and malignant tumors were found to be lymphangioma and non-Hodgkin lymphoma, respectively. Although benign tumors in the head and neck region was more prevalent than malignant tumors, a low rate of benign tumors in males indicate that more attention should be paid to the diagnosis as early as possible for proper management of the condition.
